# The protective effect of vitamin E on rats' ovarian follicles following an administration of diazinon: An experimental study

**DOI:** 10.18502/ijrm.v17i2.3985

**Published:** 2019-03-20

**Authors:** Zinat Sargazi, Mohammad Reza Nikravesh, Mehdi Jalali, Hamid Reza Sadeghnia, Fatemeh Rahimi Anbarkeh

**Affiliations:** ^1^Department of Basic Sciences, Torbat Heydariyeh University of Medical Sciences, Torbat Heydariyeh, Iran.; ^2^Department of Anatomy and Cell Biology, School of Medicine, Mashhad University of Medical Sciences, Mashhad, Iran.; ^3^Department of Pharmacology, Faculty of Medicine, Mashhad University of Medical Sciences, Mashhad, Iran.

**Keywords:** *Diazinon*, *Proliferation*, *Ovary*, *Vitamin E*, *Rat.*

## Abstract

**Background:**

Diazinon (DZN) is an organophosphate insecticide that has been widely utilized in agriculture all over the world and caused many negative effects on different species such as plants and animal species, especially on a human.

**Objective:**

The aim of the present study was to evaluate the protective effect of vitamin E on rats' ovarian follicles following an administration of diazinon.

**Materials and Methods:**

A total of 30 adult female Wistar rats were divided into five groups: a control group (without any intervention), sham group (received only pure olive oil, as solvent), experimental group I (DZN+olive oil, 60 mg/kg), experimental group II (vitamin E, 200 mg/kg), and experimental group III (DZN: 60 mg/kg+vitamin E: 200 mg/kg). All drugs were injected intraperitoneally, except vitamin E which was administrated by gavage. The animals were scarified after two weeks and left ovary was used to measure proliferation of ovarian follicles. Tissues were analyzed by the PCNA technique and viewed with an optical microscope for evaluating cells proliferation.

**Results:**

The result of the present study revealed that the number of proliferative cells in the experimental group I decreased significantly in contrast to the control group in secondary and Graffian follicles (p< 0.001). The administration of vitamin E plus DZN significantly increased proliferative cells compared to the DZN group (p< 0.001). Primordial follicles showed that all study groups were lacking PCNA positive cells, which means no expression of PCNA in these follicles. The results of this study showed that primary follicles in all study groups had a few and scattered PCNA positive cells with no significant difference between the groups (p> 0.05).

**Conclusion:**

Results showed that DZN reduced proliferation in secondary and Graffian follicles and vitamin E increased it. The results of this study suggested that vitamin E by its antioxidant activity was able to improve the DZN-induced ovarian toxicity.

## 1. Introduction

Diazinon (0, 0-diethyl-0-[2-isopropyl-6-methyl-4-pyrimidinyl] phosphorothioate is one such broad-spectrum organophosphate (OP) insecticide used to control a variety of insects in agriculture and domestic applications (1). Despite its low resistance in the environment, it can be highly toxic for animals and humans (2). They have been utilized in industry, agriculture, farming, medicine, animal keeping, and households to kill nematodes fungi, insects, worms, and weeds for five decades (3–5). Unfortunately, the indiscriminate use of DZN harm different species in the environment and lead to persistence which is an enhancing concern in this field (6, 7). amounts of OP insecticides remains in an environment including in soil, the tissue of organisms, vegetables, grains, and other food products (7). These pesticides are absorbed by the body through the skin and mucous membranes via oral cavity and inhalation (8).

Diazinon negatively affects several organs of the body including liver, kidneys, pancreas, immune system, urinary and reproductive systems, and cardiac and vascular walls, and induce hematological and biochemical changes. Previous studies have shown that insecticides (permethrin) induced testicular damages, which exhibits histological alterations in adult male testes (9). Ovary which has a main role in synthesizing hormones and producing oocyte reproductive function is One of the affected organs (10, 11). The main mechanism of action of DZN is inhibiting acetyl-cholinesterase (AChE) activity in the target tissues including ovary (3, 7). Additionally, it can increase the formation of Reactive Oxygen Species (ROS) and so induce oxidative stress in the body of mammals and other organisms (12, 13).

Chronic poisoning with OPS is more than just AChE inhibition and leads to inducing apoptosis by activating internal and external pathways, hence, in previous study the induction of apoptosis in different body tissues has been accessed via the activation of the caspases by OPs (14) The measurement of the DNA synthesis is a sign of cell proliferation. In most cases, passing the cell from G1 phase to S phase is the basic step for entering the cell division, in other words, the cell division is adjusted in this step. Proliferation Cell Nuclear Antigen (PCNA) is a non-histone protein that has an auxiliary role for DNA polymerase delta. PCNA expression has a direct relationship with mitotic activity, so it can be used as an indication of cell proliferation (15–17). Antioxidants detoxify excessive ROS and have a basic role in maintaining oxidant/antioxidant balance in the body. Antioxidants are of two types: enzymatic and non-enzymatic (12). Vitamin E (α-tocopherol)) is a fat-soluble antioxidant in cells and protect cellular membranes and lipoproteins from peroxidation. In addition, several studies have indicated that vitamin E has an effective role in inhibiting the free radical formation and so reduce lipid peroxidation in biological systems (5). The previous study had shown that vitamin E deficiency can lead to infertility due to its polyphenol components rapidly generates free radicals and protects sperm (18). Furthermore, antioxidants have a substantial role in the female reproductive system (19). The aim of this study was to investigate the effects of DZN, an OP insecticide, on the proliferation of ovarian follicles in adult rats and evaluate the protective role of vitamin E in rat ovarian tissue.

## 2. Materials and Methods

### Animals

In this experimental study, 30 adult female Wistar rats were divided into five groups of six rats each: control group (no intervention), sham group (only pure olive oil), experimental group I (DZN, 60 mg/kg), experimental group II (DZN60 mg/kg+vitamin E 200 mg/kg) and vitamin E-treated (vitamin E, 200 mg/kg). Olive oil was used as a solvent (20–24). DZN and solvent were administrated by intraperitoneal injection and vitamin E was given through gavage. All of these animals were scarified after two weeks and ovaries were used to measure proliferation of ovarian follicles. The rats were fed a standard chow and water ad libitum, and exposed to a 12-h light/dark cycle, at a temperature of 22${}^{\circ }$C. All the experimental protocols were approved by the Ethical Committee of the Mashhad University of Medical Science.

### Chemicals

For this study, technical DZN 98% was purchased from Ariashimi Company. DZN was dissolved in olive oil, vitamin E (α-tocopherol acetate) was purchased from Sigma. Proliferation in tissue was done by PCNA kit (Zymed company).

### Histological analysis

Tissue samples were fixed in paraformaldehyde (4%), solved in phosphate buffer saline (PBS) (100 mL) for 14 to 16 h, dehydrated in ascending grades (20–100%) of alcohol for 45 min to 1 h, and then cleared in alcohol-xylene (50:50) and xylene (three times). Tissues were then fixed in paraffin; samples were cut in 5 μm sections with a microtome and placed on poly L-lysine slides. Slides were deparaffinized and hydrated in descending grades of alcohol. Tissues were analyzed by the PCNA technique and viewed with an optical microscope.

Primary Follicles were defined if had a single layer of cuboidal granulosa cells; preantral Follicles if had one or two small spaces filled with follicular fluid; If had a single large antral space were antral; if oocyte was surrounded by a rim of cumulus cells were defined preovulatory; atretic if follicles were deformed or oocyte was absent or pyknotic nuclei was present in granulosa cells; and corpora lutea. Types of follicles were counted in each ovary (25).

### PCNA immunohistochemical technique

Part of the serial sections obtained from each sample was evaluated for cell proliferation phenomenon in ovarian follicles. This evaluation was performed by the immunohistochemistry method based on the use of PCNA antibodies. (Kit of PCNA was purchased from Zymed Company.) After deparaffinizing and hydrating in alcohol with descending grades, samples were washed thrice with a PBS solution, and then peroxidase activity of tissue was blocked by H2O2 3% in Methyl alcohol. After that, the blocking solutions were removed from the sections and slides were incubated for 30 min with Biotin primary antibodies against PCNA. In the next stages, the sections were incubated with DAB (Diaminobenzidine) and Streptavidin-peroxidase. The samples were washed with PBS and later with distilled water, dehydration was performed with ascending grades of ethylic-alcohol, and clearing was done by xylene. Finally, samples were mounted with special glue and were ready for study with optical microscopy for evaluating cells proliferation. The PCNA positive cells would appear in Brown (26).

### Stereology technique

#### Quantification of PCNA-positive cells

The sections were scanned and were photographed using a light microscope (UPlan FI, Japan) were used for scanning and photographing the sections. Morphmetrical methods were used to count PCNA positive cells per unit area in the ovary. The number of PCNA positive cells were counted using grades Unbiased frames. The mean number of PCNA positive cells per unit area (NA) in different types of ovarian follicles in different groups of rats was calculated using the following formula (27):

ΣQ

a/f.ΣP.

`ΣQ' is the sum of counted particles appearing in sections, `ΣP' is the sum of frame associated points hitting space and `a/f' is the area associated with each frame.

### Statistical analysis

Data were analyzed using SPSS 16 software (Chicago, IL, USA). Results are expressed as mean±SD. Statistical analysis was performed using ONE-WAY ANOVA, followed by Tukey test to compare the differences between means. Differences were considered statistically significant at p< 0.05.

## 3. Results

### Effect of DZN and vitamin E on cell proliferation in ovarian follicles

The results based on the types of ovarian follicles are as follows:

### Primordial follicles

In all five groups including control, sham, experimental group I, experimental group II, and vitamin E-treated group, the follicles were free of PCNA-positive cells and no expression of PCNA was observed in these follicles.

### Primary follicles

In these follicles, a few and scattered PCNA-positive cells were observed in all groups. Statistical analysis showed no significant difference between the groups (p> 0.05).

### Secondary follicles

**Figure 1:**
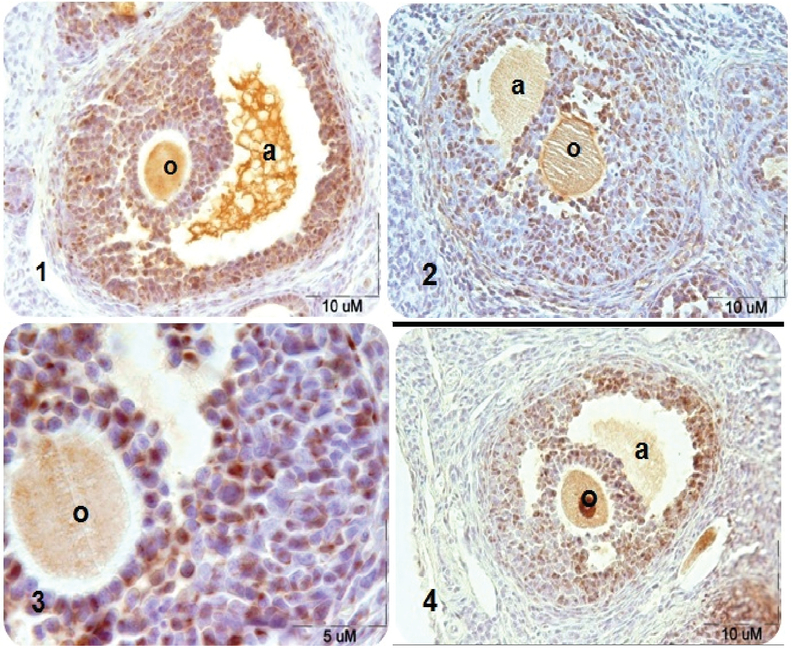
Microscopic sections of rat's secondary ovarian follicles after PCNA immunohistochemical technique. PCNA-positive nuclei are seen in brown- (1) control group, (2) DZN-treated group in which DZN decreased the PCNA-positive cells significantly compared to the control group, (3) DZN-treated group by magnification ×100, and (4) DZN+vitamin E-treated group in which the number of PCNA-positive cells increased significantly compared to the DZN group. Note: a: antrum; o: oocyte.

**Figure 2:**
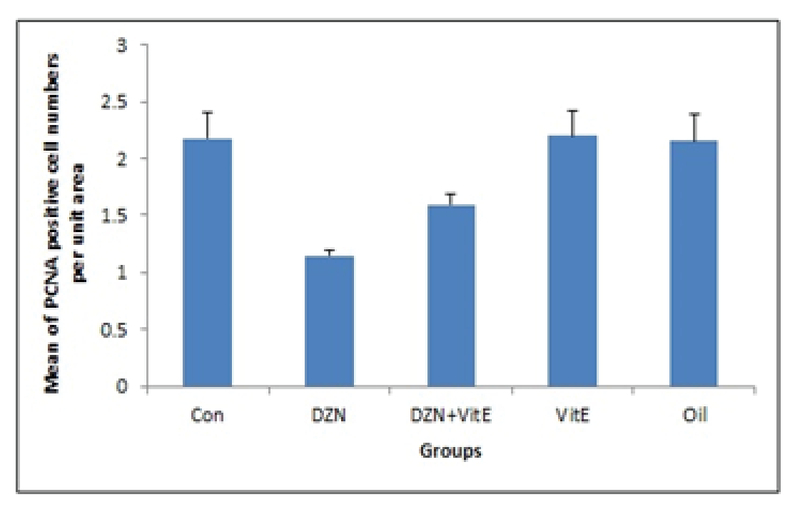
Comparison of proliferating cell per unit area in secondary follicles in different groups (mean±SD). Note: ${}^{***}$p< 0.001 vs. control; ${}^{\#\#\#}$p< 0.001 vs. DZN; Con: control group; DZN: diazinon group; Vit E: vitamin group.

According to Figure 1 and 2, the number of proliferating cells decreased significantly in the DZN-treated group compared to the control group (p< 0.001). The least amount of proliferating cells was observed in DZN-treated group. In the experimental group II (vitamin E +DZN-treated group), the number of proliferating cells increased significantly compared to the diazinon group (p< 0.001). Moreover, the number of proliferating cells in sham and vitamin E-treated groups were similar to the control group, and there was no significant difference between these groups (p> 0.05).

### Graafian follicles

**Figure 3:**
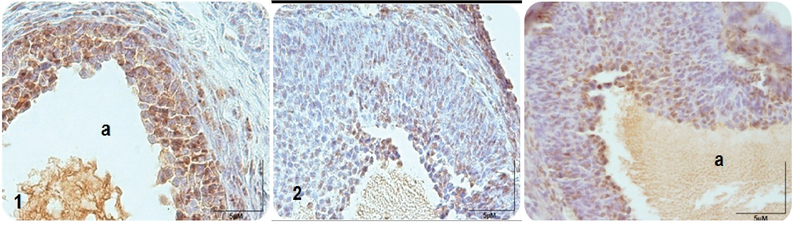
Microscopic sections of rat Graafian ovarian follicles after PCNA immunohistochemical technique. PCNA-positive nuclei are seen in brown: (1) control group, (2) DZN-treated group where DZN decreased significantly PCNA-positive cells compared to control group, and (3) DZN+vitamin E-treated group where a number of PCNA-positive cells increased significantly compared to the DZN group; a: antrum, o: oocyte.

**Figure 4:**
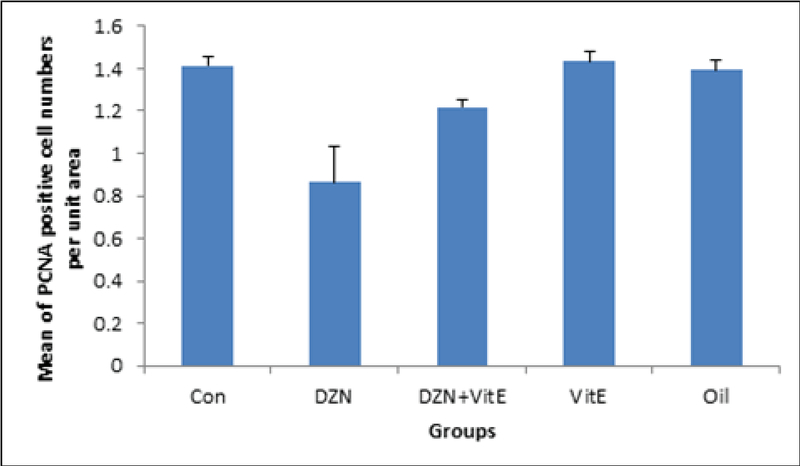
Comparison of proliferating cell per unit area in Graafian ovarian follicles in different groups (mean±SD); ${}^{***}$p< 0.001 Diazinon compared with the control group, ${}^{**}$p< 0.01 Diazinon+vitamin E group compared with the control group, and ${}^{\#\#\#}$p< 0.001 vs. DZN.

According to Figure 3 and 4, the number of proliferating cells in sham and vitamin E-treated groups was similar to the control group, and there was no significant difference between these groups (p> 0.05). However, the number of proliferating cells were significantly higher in sham and vitamin E-treated groups compared to the experimental group I(DZN-treated group) and the experimental group II (vitamin E+DZN-treated group) (p< 0.001). The least amount of PCNA-positive cells was observed in the DZN-treated group. The number of proliferating cells decreased significantly in diazinon group compared to the control group (p< 0.001). The number of PCNA-positive cells increased in the experimental group II (vitamin E+DZN-treated group) compared to the experimental group I(DZN-treated group) (p< 0.001).

## 4. Discussion

Diazinon is one of organophosphorus pesticide that is not only used in pest control of vegetables and fruits, but it is also used as an ectoparasiticide for cattle in veterinary utilizations. After absorption from the digestive tract, DZN is rapidly metabolized in the body. Several organs such as the liver, pancreas, kidney, immune system, cardiac and vascular wall and reproductive system have biochemical and histopathological changes by DZN (28, 29). The ovary is one of the target organs which has a basic role in the reproductive performance by synthesizing hormones and production oocyte (11, 30, 31). This study was performed to study the effects of DZN on the proliferation of ovarian follicles in adult female rats and to evaluate the protective role of vitamin E. The results of this study demonstrated that DZN decreased the number of proliferating cells in ovarian follicles. This study was performed to study the effects of DZN on the proliferation of ovarian follicles in adult female rats and to evaluate the protective role of vitamin E. The results of this study demonstrated that DZN decreased the number of proliferating cells in ovarian follicles. Furthermore, vitamin E improve the toxic effect of DZN by its antioxidant activity. The inhibition of acetylcholinesterase (AChE) activity in the target tissues is the most important action of DZN compound toxicity (3). AChE is an enzyme that catalyzes acetylcholine and prevents its accumulation at cholinergic synapses (2, 5).

Diazinon applies its toxic effect by binding its oxygen analog to the neural enzyme AChE, leading to accumulation of endogenous acetylcholine on neural tissue and target organs (3). Also, DZN causes the formation of ROS and induces oxidative stress. Mitochondria play important role in apoptosis. Mitochondrial dysfunction by oxidative stress release cytochrome C and activate caspase that leads to apoptotic cell death (6). Previous studies had shown the impact of OP pesticides on cell proliferation in different tissues. Measurement of DNA synthesis is a sign of cell proliferation (15–17). Cell passage from G1 phase to the S phase is the basic step for entering cell division and cell division is adjusted in this step. Proliferating cell nuclear antigen (PCNA) is a non-histone protein that has an auxiliary role for DNA polymerase delta. PCNA expression has a direct relationship with mitotic activity, so it can be used, as an indication of cell proliferation. One of the known functions of PCNA is processing Delta and Epsilon DNA Polymerases. PCNA also interacts with proteins involved in cell cycle and play a role in cell cycle control through interactions with CdkCycline complex. PCNA expression has a direct relation with mitotic activity, therefore it can be used as a marker for cell proliferation in ovarian follicles. This is the first study that evaluated the effects of DZN on the proliferation of follicles in the ovary of rats and the protective role of vitamin E in this regard. In the present study, the evaluation of proliferating cells by PCNA technique on primordial follicles showed that all study groups were lacking PCNA-positive cells, which means no expression of PCNA in these follicles.

According to the previous studies and the results of this research regarding the lack of PCNA expression in the follicles, it is probable that the concentration and primary increase of Granulosa cells in the early stages of follicular growth be the result of the adjacent cells convergence to the ovarian stroma. Furthermore, the results of this study showed that primary follicles in all study groups had a few and scattered PCNA-positive cells with no significant difference between groups. The result of this study also demonstrated that the number of proliferating cells in secondary and Graafian follicles decreased significantly in diazinon-treated group compared with the control group. In addition, in this study, the utilization of vitamin E plus diazinon increased significantly the number of proliferating cells in this follicles as compared to the diazinon group. This result proves that antioxidants such as vitamin E improve cells' antioxidant defense system or protect cells from oxidative stress. The decrease in cell proliferation due to diazinon exposure confirmed that OPs through the production of free radicals and oxidative stress damage DNA. The natural response to DNA damage is the activation of checkpoints that stop the cell cycle and repair the damage, and cause aging or apoptosis (32, 33). Colovic and colleagues demonstrated that different doses of diazinon decreased proliferation of lymphocytes and human-derm fibroblasts in a culture that this reduction was dose-dependent (34). Videau and co-workers. reported that malathion can reduce sperm count and increase sperm abnormalities in shape and motility (35).

## 5. Conclusion

DZN reduces the number of proliferating cells in secondary and Graafian follicles. Vitamin E by its antioxidant activity was able to improve the toxic effect of DZN. Therefore, vitamin E can protect ovarian tissues against this toxicity.

##  Conflict of Interest 

The authors declare that there is no conflict of interest.
